# Combined Treatment of Uterine Arteriovenous Malformation Using a 16 Fr Miniresectoscope in an Office Setting Without Anesthesia: A Case Report

**DOI:** 10.1155/2024/9216109

**Published:** 2024-07-30

**Authors:** Giovanni Lipari, Alessandro Messina, Carolina Teston, Paolo Alessi, Alessia Mariani, Tiziana Bruno, Fernanda Florio, Sofia Vegro, Livio Leo, Bianca Masturzo

**Affiliations:** ^1^ Division of Obstetrics and Gynecology Department of Maternal Neonatal and Infant Medicine University Hospital “Degli Infermi”, Ponderano, Italy; ^2^ Department of Obstetrics and Gynecology Hospital Beauregard, AUSL Valleè d'Aoste, Aosta, Italy

**Keywords:** hysteroscopy, miniresectoscope, nitrous oxide, office hysteroscopy, pericervical anesthesia, uterine arteriovenous malformation

## Abstract

Arteriovenous malformations (AVMs) are abnormal connections between arteries and veins that bypass the capillary system. Among AVMs, uterine ones are very rare, and it is not possible to have clear data on their incidence, as a good part of the patients remain clinically asymptomatic. Uterine AVMs consist of abnormal communications between branches of the uterine artery and the myometrial venous plexus. They can lead to significant bleeding, resulting in severe anemia and the need for transfusions. Both medical and surgical therapeutic approaches are described in the literature; as regards surgical treatments, the hysteroscopic excision of the endometrial mass represents a conservative and minimally invasive approach. However, there are no reported cases in the literature of AVMs treated using a hysteroscopic approach under local anesthesia and in an office setting. In this article, we propose the case of a young woman diagnosed with postpartum uterine AVM, treated using a 16 Fr miniresectoscope (GUBBINI system; Tontarra Medizintechnik®, Tuttlingen, Germany) in an office setting with a pain control protocol (pericervical infiltration and nitrous oxide via bucconasal mask). No complications occurred, and the woman was discharged immediately after the procedure. Finally, the patient was asked how tolerable and acceptable the procedure was compared to expectations; the woman defined the procedure as very bearable and well tolerated. The outpatient treatment, with an adequate pain control protocol, proved to be less invasive for the woman, did not require narcosis and hospital admission, but was equally therapeutic and effective compared to the treatment performed in the operating room.

## 1. Introduction

Arteriovenous malformations (AVMs) are abnormal connections between arteries and veins that bypass the capillary system. Peripheral onset at the uterine level is rare but represents one of the main causes of abnormal vaginal bleeding. Uterine AVMs consist of abnormal communications between branches of the uterine artery and the myometrial venous plexus. They can lead to significant bleeding, resulting in severe anemia and the need for transfusions [1].

They represent 1%–2% of all genital or intraperitoneal hemorrhages, but it is not possible to have a precise estimate of the incidence because many cases remain undiagnosed because they are asymptomatic. The incidence of this pathology has significantly increased in recent years, both due to improvements in diagnostic techniques and, above all, as a result of an increase in uterine interventions (cesarean sections, abortions, curettages, etc.) [2]. The etiology is not clear, but uterine traumas seem to play an important role. The abnormal connection between arteries and veins typically forms during the healing process. It is estimated that the incidence is 0.63% higher following childbirth or abortion [3]. Prevalence can vary from less than 1% to about 3% of women of reproductive age, depending on the studies and populations examined.

They can be classified as congenital or acquired AVMs, which predominantly occur following trauma, especially surgical trauma. However, nonsurgical causes of onset are also described, such as infections, trophoblastic disease, or malignant tumors involving the uterus [4].

The diagnosis of this condition is possible with the help of ultrasound, and using color Doppler, it is possible to estimate the severity by evaluating the PSV (peak systolic velocity) within the involved branch of the uterine artery. If the PSV is < 40 cm/s, a wait-and-see approach can be considered, as the risk of bleeding is low. If PSV is > 40 cm/s, it is preferable to opt for therapy, which can be medical or surgical [5, 6].

As regards medical therapy, various drugs can be used, and in particular, the use of progestins has been shown to be effective in resolving uterine bleeding in about 90% of postabortion patients [7].

In more severe cases, conservative therapy can be considered through selective embolization of the involved branches of the uterine artery. These branches are reached through a catheter inserted into the femoral artery. The procedure is performed under angiography guidance with a contrast dye that helps the radiologist identify vessels. Various agents can be used, either alone or in combination, in particular glue, gelfoam, coils, polyvinyl alcohol (PVA), or microspheres [1].

This represents first-line therapy for fertile women with recurrent vaginal bleeding, severe anemia, or hemodynamic instability who want to preserve fertility [8]. There are no reported cases of infertility or intrauterine growth restriction in subsequent pregnancies of patients undergoing embolization.

A recent systematic review [9] examined 95 studies, totaling 371 patients treated with embolization, highlighting a procedure success rate of 88.4% and a low complication rate (1.8%). Seventy-seven patients became pregnant following the procedure, with 77% of them showing no obstetric problems. Another systematic review and meta-analysis, even more recent [10], reported 44 pregnancies in 189 analyzed patients, with a live birth rate of 88.6%, comparable to that of the general population.

Surgical therapy with hysterectomy is an option for postmenopausal women who do not need to preserve fertility or, in severe cases, after the failure of conservative therapy [8]. Other therapeutic strategies are reported in the literature but are rarely used, such as laparoscopic occlusion of the internal iliac arteries [11] or unilateral ligation of the uterine artery and ovarian ligament [12].

Another possibility for conservative and minimally invasive therapy is represented by the hysteroscopic excision of the endometrial mass, as reported in a 2019 case report [13] of a 20-year-old woman. The procedure was performed in the operating room, with a total duration of 1 h and 20 min, and the patient was discharged at home 7 days after the procedure.

Other authors [14] report 11 cases of AVMs treated using hysteroscopy under general anesthesia, with average procedure durations of about 30 min, without the need for hospitalization > 24 h, and without the onset of complications. Six out of 11 women became pregnant within 1 year of the procedure, and five of them completed the pregnancy without complications.

There are no reported cases in the literature of AVMs treated using a hysteroscopic approach under local anesthesia and in an office setting.

Based on these premises, in this article, we propose the case of a young woman diagnosed with postpartum uterine AVM, treated using a 16 Fr miniresectoscope in an office setting with a pain control protocol (pericervical infiltration and nitrous oxide via bucconasal mask).

## 2. Methods

A young 40-year-old woman with a previous cesarean section in her medical history was referred to our center after a diagnosis of uterine AVM after a second cesarean section performed in August 2023.

The patient was in good health, without any comorbidities to report or ongoing chronic therapies.

**Figure 1:**
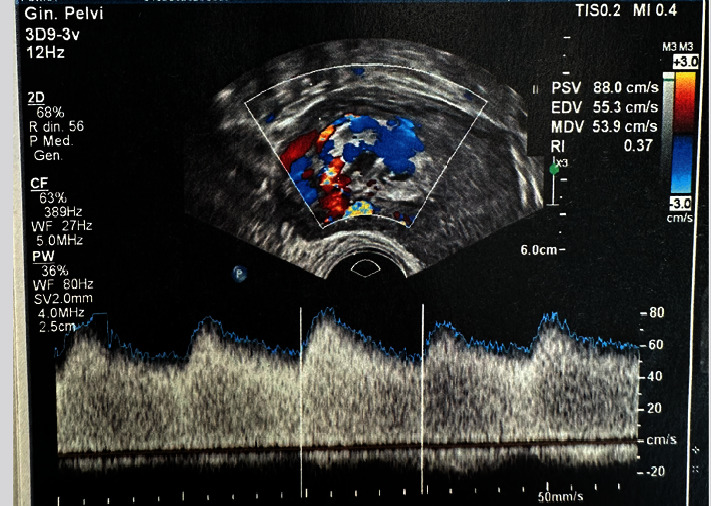
Transvaginal ultrasound with evidence of a nonhomogeneous mosaic area of the anterofundal wall with vascularization Colorscore 4 and PSV 88 cm/s (diagnosis of uterine arteriovenous malformation).

The woman, with persistent blood loss in the puerperium, underwent a transvaginal ultrasound with evidence of a nonhomogeneous mosaic area of 15 × 22 mm of the anterofundal uterine wall with high vascularization (Colorscore 4 and PSV 88 cm/s—diagnosis of uterine AVM) (Figure 1).

After the diagnosis, the patient first underwent embolization of the uterine arteries, and, subsequently, whereas the woman continued to be significantly symptomatic, a second transvaginal ultrasound check was performed (PSV 65.3 cm/s); for these reasons, we decided to complete the treatment with a 16 Fr miniresectoscope (GUBBINI system; Tontarra Medizintechnik®, Tuttlingen, Germany) in an outpatient setting.

In view of the hysteroscopic treatment and considering that the woman was still breastfeeding, continuous progestin therapy with drospirenone 4 mg was prescribed.

The hysteroscopic treatment was performed using a saline solution as a distension medium, and with adequate pain control, the procedure was performed in an office setting with dim lights and relaxing music. To render the woman more relaxed, a nitrous oxide in a bucconasal mask was administered 2–3 min before the start of the surgery, and a pericervical infiltration with mepivacaine 2% at 3 and 9 o'clock of the external uterine orifice was performed. After pericervical analgesia, nitrous oxide was no longer necessary.

A vaginoscopic approach was used; for the resection of the AVM, a 90° angled bipolar cutting loop was used.

It was not necessary to coagulate the implant base, and no bleeding occurred.

The surgery lasted 12 min, and there were no complications.

**Figure 2:**
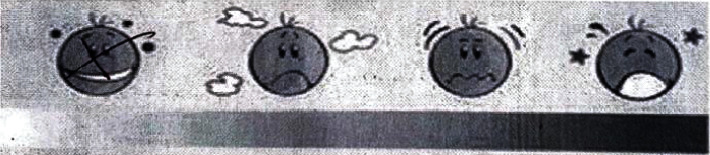
The woman defined the procedure as very bearable and well tolerated.

**Figure 3:**
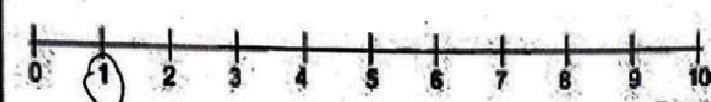
VAS (Visual Analogue Scale) from 1 to 10: the patient reported a pain level of 1.

At the end of the procedure, the patient was asked how tolerable and acceptable the procedure was compared to expectations; the woman defined the procedure as very bearable and well tolerated. We asked the woman to communicate the pain felt during hysteroscopy using the VAS (Visual Analogue Scale) scale from 1 to 10, and the patient reported a pain level of 1 (Figures 2 and 3).

The patient was immediately discharged, and progestin therapy was suspended. The woman no longer reported blood loss for a few days after the operation.

Histological analysis confirmed the diagnosis of involuted placental residues in the myometrium.

**Figure 4:**
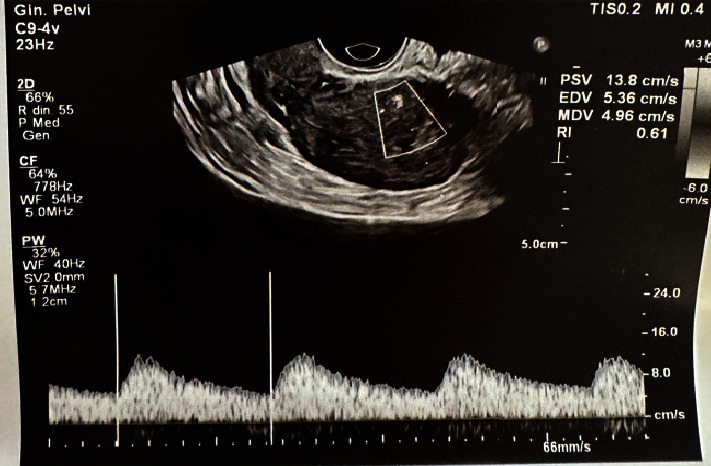
Transvaginal ultrasound check 30 days after hysteroscopy, which highlighted a PSV of 13.8 cm/s.

The woman, completely asymptomatic from a clinical point of view (without any intermenstrual bleeding), finally performed a transvaginal ultrasound check 30 days after hysteroscopy, which highlighted a PSV of 13.8 cm/s and therefore a resolution of the pathological condition (Figure 4).

## 3. Discussion

Although peripheral onset at the uterine level is rare, AVMs still represent one of the main causes of abnormal vaginal bleeding, and they are particularly complicated to treat as there remains a great heterogeneity of data regarding treatment options and their outcomes.

Various surgical approaches to this pathology are proposed in the literature; among these, it is important to underline the relevance of the hysteroscopic approach, as it is conservative and minimally invasive. However, in the literature, there are no reported cases of AVMs treated with a hysteroscopic approach in an outpatient setting and with local anesthesia, but all reported cases were treated in the operating room with long operating times and performed under general anesthesia.

The surgical technique that we propose in this article combines all the benefits of miniaturised instruments and bipolar energy.

In fact, with the 16 Fr miniresectoscope (GUBBINI system; Tontarra Medizintechnik®, Tuttlingen, Germany), we entered the uterine cavity directly with a vaginoscopic approach, without cervical dilatation. We used a 90° angled bipolar cutting loop to completely resect the nonhomogeneous mosaic area of the anterofundal uterine wall with high vascularization.

In order to further improve surgical performance in an outpatient setting, pain control is essential. We actually performed hysteroscopic treatment using nitrous oxide via bucconasal mask 2–3 min before starting hysteroscopy, then performed pericervical infiltration with mepivacaine 2%; after pericervical anesthesia, nitrous oxide was no longer administered.

As previously described, at the end of the procedure, we asked the woman to communicate the pain felt during hysteroscopy using the VAS from 1 to 10 and to tell us if the pain experienced during the operation was less or more than expected. The level of pain (VAS 1) and the high tolerability of the procedure reported by the patient highlight how this type of approach was successful.

Therefore, we believe that this kind of treatment, performed with miniaturised instruments in an office setting with adequate pain control, can be considered a valid option in the surgical treatment of uterine AVM, in particular, due to its minimal invasiveness and great advantages for the patient.

In our case, the postoperative outcome was assessed on a transvaginal ultrasound check 30 days after hysteroscopy, which highlighted a PSV of 13.8 cm/s and, finally, a resolution of the pathological condition.

## 4. Conclusions

With this case report, we would like to propose an innovative treatment, less invasive for women and less expensive, for postpartum uterine AVM. We have actually described the case of a young woman diagnosed with postpartum uterine AVM treated with a 16 Fr miniresectoscope in an outpatient setting.

The outpatient treatment, with an adequate pain control protocol, proved to be less invasive for the woman, did not require narcosis and hospital admission, but was equally therapeutic and effective compared to the treatment performed in the operating room.

However, further literature data and further case series are needed before it can be defined as a routine treatment.

## Data Availability

The data that support the findings of this study are available from the corresponding author upon reasonable request.
